# Can We Predict Graft Intolerance Syndrome After Kidney Transplant Failure? External Validation of a Previously Developed Model

**DOI:** 10.3389/ti.2023.11147

**Published:** 2023-05-05

**Authors:** Kim Bunthof, Khalid Saboerali, Jacqueline Van De Wetering, Azam Nurmohamed, Frederike Bemelman, Arjan Van Zuilen, Jan Van Den Brand, Marije Baas, Luuk Hilbrands

**Affiliations:** ^1^ Department of Nephrology, Radboud University Medical Centre, Nijmegen, Netherlands; ^2^ Department of Internal Medicine, Bravis Ziekenhuis, Roosendaal, Netherlands; ^3^ Department of Nephrology, Amsterdam University Medical Center, Amsterdam, Netherlands; ^4^ Department of Nephrology, Erasmus Medical Center, Rotterdam, Netherlands; ^5^ Department of Nephrology and Hypertension, University Medical Center Utrecht, Utrecht, Netherlands; ^6^ Research Suite, Erasmus Medical Center, Rotterdam, Netherlands

**Keywords:** prediction model, graft intolerance syndrome, kidney graft failure, external validation, graft nephrectomy

## Abstract

Previously we established a prediction model for graft intolerance syndrome requiring graft nephrectomy in patients with late kidney graft failure. The aim of this study is to determine generalizability of this model in an independent cohort. The validation cohort included patients with late kidney graft failure between 2008 and 2018. Primary outcome is the prognostic performance of our model, expressed as the area under the receiver operating characteristic curve (ROC-AUC), in the validation cohort. In 63 of 580 patients (10.9%) a graft nephrectomy was performed because of graft intolerance. The original model, which included donor age, graft survival and number of acute rejections, performed poorly in the validation cohort (ROC-AUC 0.61). After retraining of the model using recipient age at graft failure instead of donor age, the model had an average ROC-AUC of 0.70 in the original cohort and of 0.69 in the validation cohort. Our original model did not accurately predict the graft intolerance syndrome in a validation cohort. However, a retrained model including recipient age at graft failure instead of donor age performed moderately well in both the development and validation cohort enabling identification of patients with the highest and lowest risk of graft intolerance syndrome.

## Graphical Abstract

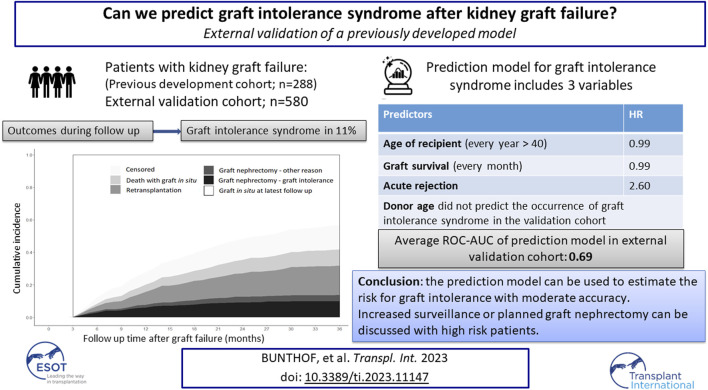

## Introduction

Although kidney graft survival has improved over the last decades, recent data indicate that the incidence of kidney graft failure within 5 years after transplantation is still 12% and 20% for living and deceased donor kidneys, respectively (1, 2). After reinstitution of dialysis, the failed graft can be removed or left *in situ*. When to perform graft nephrectomy is controversial and often depends on local clinical practice. In general, graft nephrectomy is recommended after early graft failure (within 3–6 months) in order to avoid systemic and local effects of acute rejection. After late graft failure, the risk of acute rejection is presumably much smaller, and the graft is usually left *in situ*. However, in some cases late graft nephrectomy becomes necessary. Accepted indications for graft nephrectomy are to create space for re-transplantation, to enable immediate complete withdrawal of immunosuppression, graft malignancy, recurrent transplant pyelonephritis, and graft intolerance syndrome (3–6). Graft intolerance syndrome is characterized by the presence of pain or swelling of the graft, hematuria, fever, malaise, or refractory anemia, all in the absence of an infectious process. The syndrome is reported in 30%–50% of patients with graft failure and occurs mostly within the first year after initiation of dialysis. It reflects a chronic inflammatory state induced by the retained graft and is mostly associated with discontinuation of immunosuppression. However, also patients with (low dose) immunosuppression can present with a graft intolerance syndrome. Graft intolerance syndrome is associated with high morbidity and in most cases an urgent graft nephrectomy is required. Perioperative mortality and morbidity are substantially higher for urgent graft nephrectomy than for elective graft nephrectomy (7, 8). If the need for graft nephrectomy could be predicted, this could help clinicians in deciding to perform a pre-emptive graft nephrectomy, as a planned intervention may minimize the risk of peri-operative morbidity and mortality compared to an urgent procedure.

In a previous study we used data from a single center to develop a model to predict the need for graft nephrectomy because of graft intolerance syndrome (9). The training study cohort included 288 patients with kidney graft failure, of whom 48 (16.7%) suffered from graft intolerance syndrome requiring graft nephrectomy. We used Fine and Gray regression analysis to evaluate the association between this outcome and baseline characteristics. Our final model included donor age, number of acute rejections, and graft survival (time interval between transplantation and graft failure) as predictors. External validation of a prediction model is essential to support general applicability and implementation in clinical practice. Therefore, the aim of the present study was to determine generalizability of this prediction model for graft intolerance syndrome requiring graft nephrectomy.

## Patients and Methods

### Study Population

The validation cohort included adult patients who experienced kidney graft failure at least 6 months post kidney transplantation between 2008 and 2018, and were treated in one of the following Dutch Transplant Centers: Erasmus University Medical Center (Rotterdam), Amsterdam University Medical Centers (Amsterdam), and University Medical Center Utrecht (Utrecht). Additionally, we included patients from the Radboud university medical center (Nijmegen) who were not included in the training cohort. In general, after graft failure and start of dialysis treatment, immunosuppression was gradually tapered to zero or to low dose steroids. In all patients a watchful waiting policy was followed regarding graft nephrectomy. In- and exclusion criteria were identical to those used for the training cohort. We excluded patients with one of the following events within 3 months after graft failure: re-transplantation, graft nephrectomy, death, or loss of follow up. Patients gave informed consent for sharing data in the National Organ Transplantation Registry (NOTR). This registry includes data about all national transplantation programs and is used for quality assurance and scientific research. This study was approved by each local medical ethics committee. The trial was conducted in accordance with the Declaration of Helsinki and approved by the research ethics committee of the Radboud University Medical Center, Nijmegen (2018-4732).

### Data Collection

We collected the following data from the NOTR and local patient files: age, gender, donor age, duration of graft survival, number of acute rejection episodes, and the occurrence of graft nephrectomy after graft failure. A rejection episode was defined as the need for anti-rejection therapy with or without biopsy-proven rejection. Treatment of rejection (either biopsy-proven or clinical diagnosis) after an interval of at least 3 months without acute rejection was considered to represent a new rejection episode. Indications for graft nephrectomy were retrieved from patients’ files. Graft intolerance syndrome was defined as the presence of one or more of the following clinical criteria in the absence of another plausible explanation after routine clinical examination: pain or swelling of the graft, hematuria, fever, malaise, or refractory anemia. Follow-up ended in case of a competing event (death or re-transplantation) or when patients were lost to follow-up.

### Sample Size Calculation

There are no generally accepted approaches to estimate the sample size requirement of validation studies of risk prediction models. The number of outcome events dictates the effective sample size. Our sample size was determined by the available data from participating transplant centers, and we did not choose a sample size on statistical grounds. Limited evidence suggests that a minimum of 100 events is needed to adequately quantify the performance of an existing model in other data, but more events are preferred (10).

### Statistical Analysis

The prediction rule below was applied to the patients in the validation cohort.

Log baseline cumulative hazard ( $\ln\ln H_{0}\left(t\right))=-2.0252-32.3433t^{-2}+0.0126t^{-0.5}$

Prognostic index (PI) $=0.027\times donor\;age\left[in\;years\right]-0.011\times graft\;survival\;\left[in\;months\right]+0.336\times total\;number\;of\;rejections$

Risk of graft nephrectomy at time *t*: $R\left(t\right)={1-\exp\left[-\exp\exp\left(\ln H_{0}\left(t\right)\right)\right]}^{\exp\left(PI\right)}$

We also recalibrated our model in the validation cohort by adjusting the baseline cumulative hazard without changing predicting factors. The performance of the model, expressed as the area under the receiver operating characteristic curve (ROC-AUC), and the calibration were assessed. A visual impression of the calibration of model predictions in the validation set was obtained by plotting the observed versus predicted probabilities. Finally, we retrained the original model with recipient age at the time of graft failure instead of donor age. We used the same training data and method (Fine and Gray regression) as with the previous model which was published by Bunthof et al. (10). We externally validated the retrained model with the data collected for the present study. The full analysis scripts can be accessed on https://github.com/JanvandenBrand/tect_validate.

## Results

### Study Population

**FIGURE 1:**
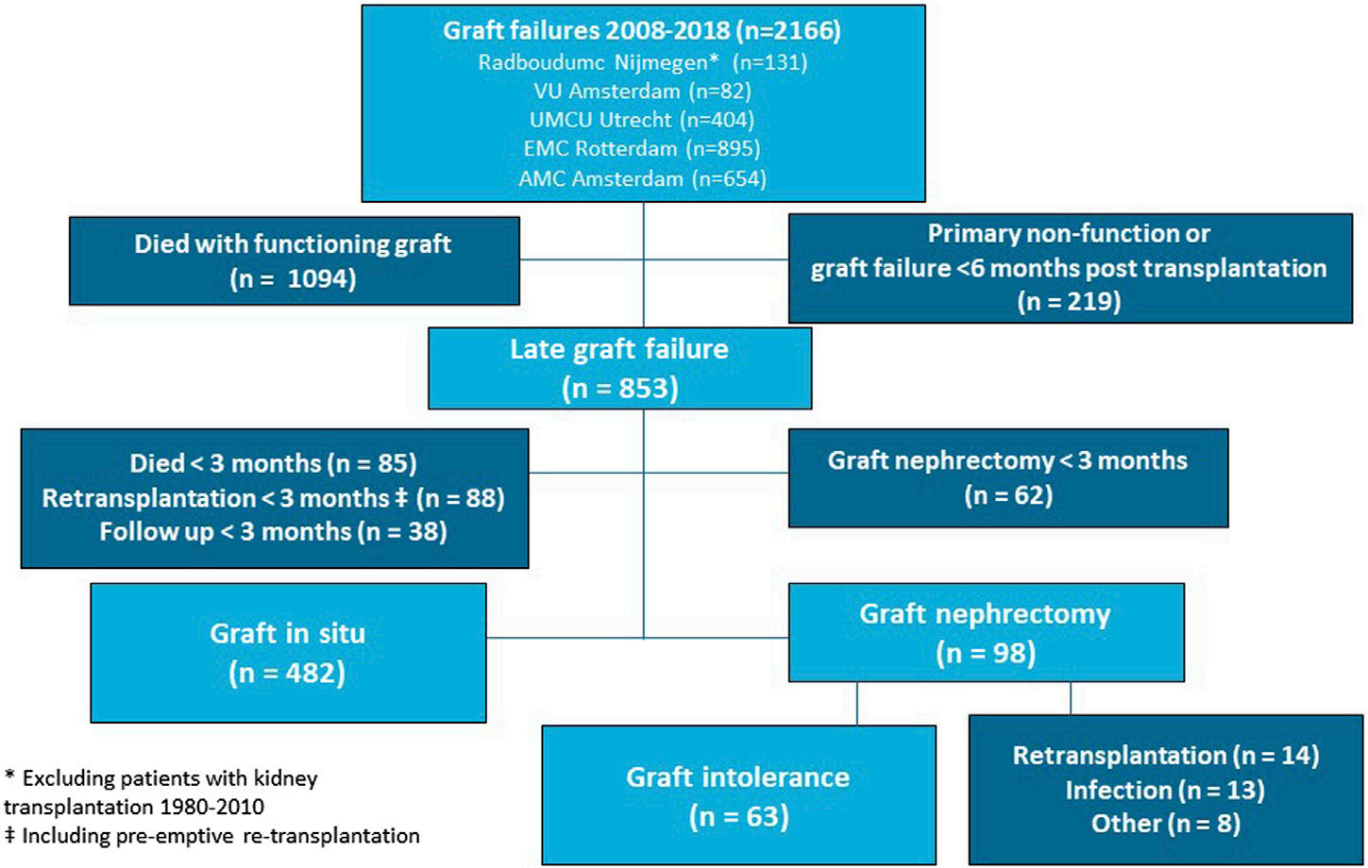
Patient inclusion.

**FIGURE 2:**
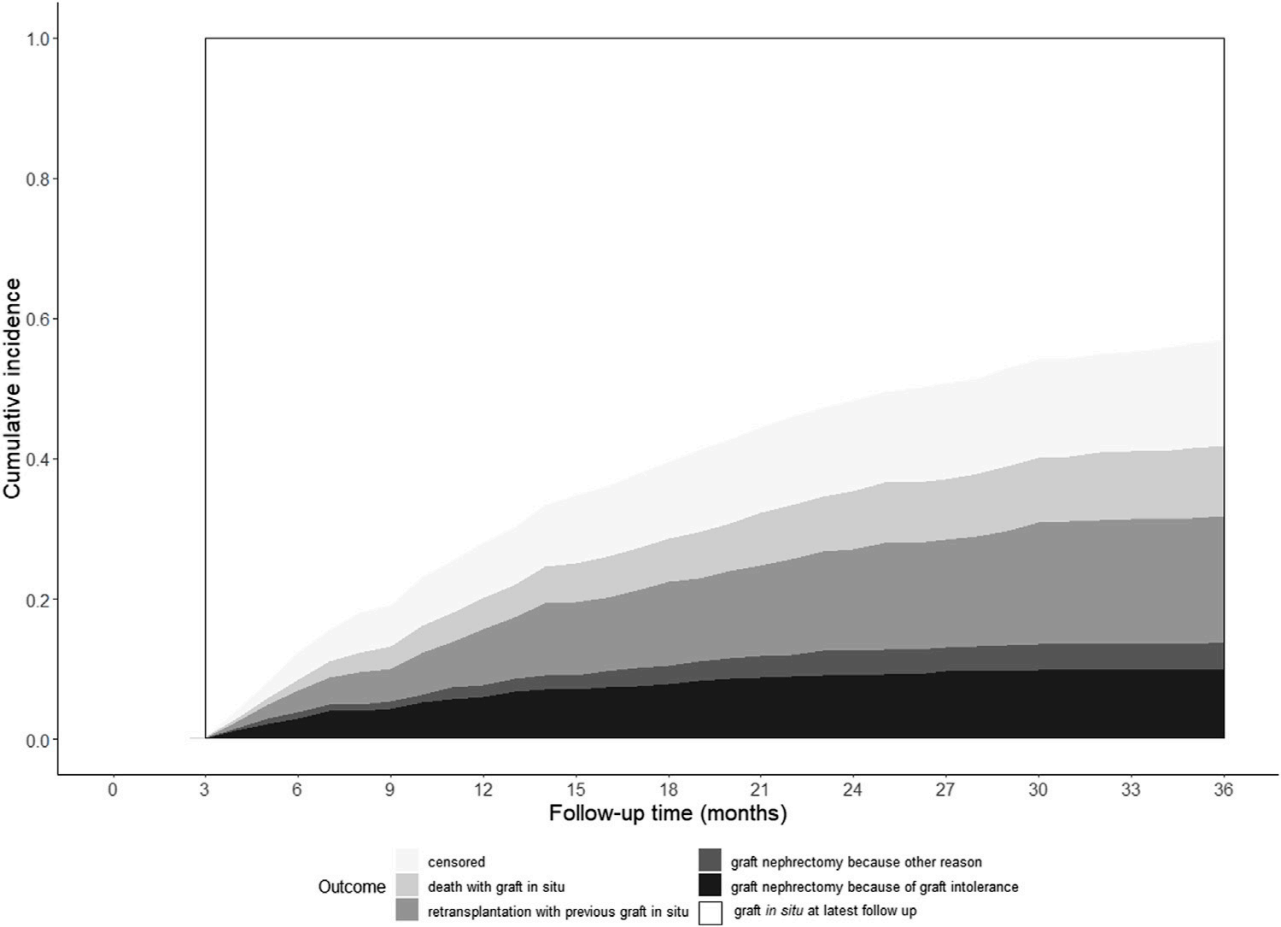
Cumulative incidence curves of study outcomes.

Our study cohort included 2,166 kidney graft failures between 2008 and 2018 (Figure 1). Patients with death as the cause of graft failure (*n* = 1,094) and patients with graft failure within 6 months after kidney transplantation (*n* = 219) were excluded from analyses. Graft nephrectomy was performed <3 months after graft failure in 62 patients and follow up ended <3 months after graft failure in 211 patients because of death, re-transplantation, or loss of follow up. Finally, we included 580 patients for validation of our model. In 98 patients of our validation cohort (16.9%) a graft nephrectomy was performed. Indications for graft nephrectomy were graft intolerance syndrome (*n* = 63), to create space for re-transplantation (*n* = 14), infection (*n* = 13), and other reasons (*n* = 8). The incidence of graft intolerance syndrome requiring a graft nephrectomy was 10.9%. Stacked cumulative incidence curves for various events during follow-up are shown in Figure 2. Patient and transplantation characteristics are shown in Table 1. Median duration of follow-up (time from graft failure to graft nephrectomy) was 10.4 and 20.1 months in patients with graft nephrectomy for graft intolerance and for other indications, respectively. In patients with a retained failed graft, median duration of follow-up (time from graft failure to death, retransplantation, or loss to follow-up) was 33 months. Compared with patients with a retained failed graft, patients with graft intolerance syndrome had a lower age at graft failure (median 43 vs. 50 years), had more acute rejection episodes, and a shorter graft survival (median 45 vs. 77 months).

**TABLE 1:** Patient and transplantation characteristics of the validation cohort.

		Graft nephrectomy		
	Allograft *in situ n* = 482	Graft intolerance n = 63	Other indication *n* = 35	p*
Patient characteristics				
Male (%)	286 (59.3)	31 (49.2)	19 (54.3)	0.13
Age at graft failure (median ± IQR)	50 (40–62)	43 (33–54)	48 (38–57)	0.001
Transplantation characteristics				
Donor age (median ± IQR)	54 (44–61)	50 (40–58)	45 (24–57)	0.09
Number of acute rejections (%)				0.05
0	237 (49.2)	23 (36.5)	12 (34.3)	
1	176 (36.5)	29 (46.0)	21 (60.0)	
2	61 (12.7)	9 (14.3)	2 (5.7)	
>2	8 (1.7)	2 (3.2)	0	
Graft survival in months (median ± IQR)	77 (43–136)	45 (26–70)	108 (50–144)	<0.001
Follow up time in months (median ± IQR)	33 (12–59)	10.4 (5.7–19.0)	20.1 (10.5–39.8)	<0.001
Center (%)				0.005
AMC, Amsterdam	149 (30.9)	24 (38.1)	10 (28.6)	
Erasmusmc, Rotterdam	233 (48.3)	22 (34.9)	15 (42.9)	
Radboudumc, Nijmegen	16 (3.3)	0	1 (2.9)	
UMCU, Utrecht	70 (14.5)	10 (15.9)	6 (17.1)	
VU, Amsterdam	14 (2.9)	14 (3.0)	3 (8.6)	

### Prediction Model

**FIGURE 3:**
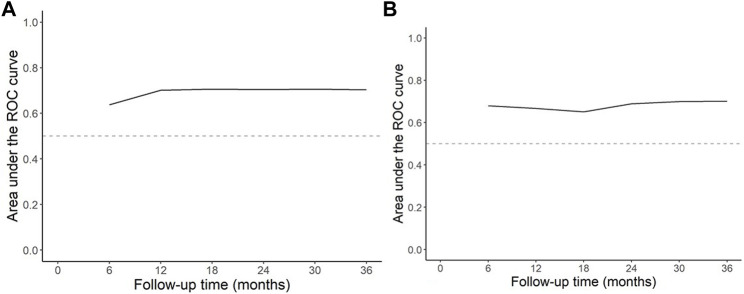
AUC of ROC curve by follow up time **(A)** retrained model in the development cohort **(B)** retrained model in the validation cohort. The above figures show the ROC-AUC estimates at various time points during follow-up. The discriminative performance is reasonably constant throughout follow-up with an average of 0.70 in the training data, and 0.69 in the validation data.

We applied our original prediction rule on the validation cohort. The obtained ROC-AUC was only 0.61 in the validation population with a poor calibration at every time point after follow up. In both cohorts, patients with a graft intolerance syndrome were younger at graft failure as compared to patients with a retained graft. Above the age of 40 years, the risk of graft intolerance syndrome requiring a graft nephrectomy decreased linearly. In our original analysis, age at time of graft failure was a significant factor in univariate analysis with a hazard ratio for graft intolerance of 0.97 for every additional year of age. It was not included in the original prediction rule because the model with donor age performed slightly better. We retrained our prediction model by replacing donor age by the age of the recipient at the time of graft failure. In this model the risk for graft intolerance changes only for patients aged above 40 years with a decrease for every additional year of age. In addition to age at graft failure this retrained prediction model included graft survival (in months) and the occurrence of any acute rejection. Hazard ratios for these factors are shown in Table 2. The model prognostic index (PI) for our retrained model is calculated by: PI = −0.0098 (age of recipient −40) (only included if age of recipient is ≥ 40 years at time of graft failure) −0.0094 (graft survival in months) + 0.9569 (if any acute rejection occurred) The ROC-AUC of this adjusted prediction rule is on average 0.70 in the original training cohort (compared to 0.69 of the original prediction model in the training cohort) and 0.69 in the validation cohort (Figures 3A, B).

**TABLE 2:** Hazard ratios for factors included in our retrained model in our validation cohort.

	Hazard ratio	95%-confidence interval
Age of recipient (every year)	0.99	0.98–1.00 (*p* = 0.11)
Graft survival (every month)	0.99	0.98–1.00 (*p* = 0.006)
Acute rejection	2.60	1.17–5.80 (*p* = 0.02)

The model object can be downloaded from https://github.com/JanvandenBrand/tect_validate/blob/main/output/fgr_model_final.RData for integration into a local machine learning operations platform. The prediction model is also available as a mobile friendly, web based RShiny application at https://jvandenbrand.shinyapps.io/predicttect/, allowing to estimate the risk of graft nephrectomy due to graft intolerance after entering age at graft failure, graft survival, and history of acute rejection.

## Discussion

The aim of this retrospective cohort study was to validate our earlier published prediction model for the need for graft nephrectomy because of graft intolerance syndrome after graft failure. The originally developed prediction model including graft survival (in months), donor age (in years), and number of acute rejections, did not predict the occurrence of a graft nephrectomy in an external validation cohort. However, an adjusted model in which donor age was replaced by recipient age at the time of graft failure performed moderately well in both the training and validation cohorts.

Patient and transplant characteristics in patients with a graft intolerance syndrome requiring a graft nephrectomy were different from patients with a retained failed kidney graft. Patients who required a graft nephrectomy because of graft intolerance syndrome, had a shorter graft survival (median 45 months vs. 77 months) and almost 65% of them had experienced one or more acute rejection episodes. These differences were also found in our original dataset and reflect a more complicated course of the kidney transplant in patients ultimately requiring graft nephrectomy. However, unlike in our original dataset, donor age did not differ significantly and was in fact numerically lower instead of higher in the group with a graft intolerance syndrome. This may explain the poor performance of the original model in the validation cohort. The validation cohort included patients with graft failure between 2008 and 2018 with a median donor age of 53 years (IQR 42–61), while the training cohort included patients with a kidney transplantation over a time span of 3 decades (1980–2010) with a median donor age of 44 years (IQR 27–54). With increasing age of the donors over time, the discriminating potential of donor age appeared to decline. With this knowledge we reanalysed our original data and noticed that both in the training and in the validation cohort the age of the recipient at time of graft failure was lower in the group with a graft intolerance syndrome. There was a linear decrease in the incidence of graft nephrectomy above the age of 40 years. A possible explanation for this finding is that older patients have a less robust immune system, also referred to as immunosenescence (11, 12) We retrained the original model using recipient age at graft failure (for recipients >40 years) instead of donor age as predictive factor and tested this in the validation model. This resulted in an average ROC-AUC of 0.69, which is similar to the performance of this model in the original cohort.

The incidence of graft intolerance in the validation cohort was relatively low. Previous studies report variable incidence rates of 30%–50% in patients with kidney graft failure (8, 13). However, we studied a selected population by excluding patients with a short graft survival (<6 months), and patients with a graft nephrectomy within 3 months after kidney graft failure, because we would like to predict graft intolerance for patients without an obvious indication for graft removal. We also observed that the overall incidence of graft intolerance syndrome in the validation cohort was lower compared to the training cohort, while in- and exclusion criteria were similar. We hypothesize that in the more recent past immunosuppression was more often continued after graft failure in order to prevent immunisation, especially in patients who qualified for a re-transplantation, resulting in a lower incidence of the graft intolerance syndrome. Unfortunately, follow-up data on immunosuppression withdrawal after graft failure were too limited to test this hypothesis and we advocate a more systematic data collection in these patients.

Prognostic models with the aim to improve the prediction of clinical events are increasingly developed and published. External validation to confirm the reproducibility and generalizability of a prediction model for different patients was found to lack in 95% of studies on prediction models(14). We performed external validation in a large cohort of patients with kidney graft failure in a recent decade treated in different centers. An important similarity between the training and validation cohort was the ‘watchful waiting strategy’ with respect to graft nephrectomy and our prediction model of a graft intolerance syndrome is therefore clinically relevant.

A limitation of this study is the low event rate. Whereas 63 events were included, we hoped to include at least 100 events for a reliable validation of the prediction model. However, there are no absolute guidelines on the event number needed to perform an external validation and it remains uncertain whether a higher number of event rates would have resulted in a better prediction model. Another limitation of this study is its retrospective nature. The documentation of patients after kidney graft failure is usually poor. Data about the withdrawal of immunosuppressive medication and the occurrence of clinically relevant problems like the graft intolerance syndrome are generally not well recorded. Nevertheless, we are fairly sure that the large majority of patients who underwent a graft nephrectomy was identified.

Morbidity and mortality in patients with a failed kidney allograft are high (15–19). The population with kidney graft failure is very heterogeneous and evidence to guide clinicians is limited. The sole guideline on this topic is published by the British Transplant Society (BTS) and contains only weak recommendations (7). An unanswered question remains whether or not to remove the failed graft. Our model reasonably differentiates between patients with a low or high risk of a graft intolerance syndrome in our training and validation cohort s. The general policy on immunosuppressive treatment in both cohorts was to taper immunosuppression to zero or to low dose steroids. Hypothetically, continuation of more intensive immunosuppression could prevent the occurrence of graft intolerance syndrome with the need of a graft nephrectomy. However, evidence to support this hypothesis is lacking. Recent studies showed that patients still experienced rejection episodes and sensitization despite the continued use of immunosuppressants beyond the first year after transplant failure (20, 21) Prospective interventional trials are needed to compare the occurrence of graft intolerance between patients with different immunosuppressive treatment strategies. In the meantime, risks and benefits of a pre-emptive graft nephrectomy could be discussed individually with patients who have no prospect of a retransplantation in the near future and a high predicted risk of graft intolerance syndrome according to our model. Additionally an elevated risk creates awareness and can prompt more active surveillance for the possible occurrence of graft intolerance syndrome. In case of early recognition graft nephrectomy could be performed before deterioration of patients with ongoing inflammation. In conclusion, the incidence of graft intolerance syndrome in patients with late graft failure (i.e., graft survival >6 months) and an initial “watchful waiting policy” regarding graft nephrectomy was 11%. Our retrained model including recipient age at time of graft failure, the occurrence of any acute rejection during latest transplant, and graft survival in months, can be used to estimate the risk of a graft intolerance syndrome with moderate accuracy. The estimated risk can be used to discuss the indication for pre-emptive removal of a failed kidney graft in individual patients.

## Data Availability

The raw data supporting the conclusion of this article will be made available by the authors, without undue reservation.
